# *Cohnella* sp. A01 laccase: thermostable, detergent resistant, anti-environmental and industrial pollutants enzyme

**DOI:** 10.1016/j.heliyon.2019.e02543

**Published:** 2019-09-30

**Authors:** Masoomeh Shafiei, Farzaneh Afzali, Ali Asghar Karkhane, S. Mehdi Ebrahimi, Kamahldin Haghbeen, Saeed Aminzadeh

**Affiliations:** a Bioprocess Engineering Group, Institute of Industrial and Environmental Biotechnology, National Institute of Genetic Engineering and Biotechnology (NIGEB), Iran; b Institute of Medical Biotechnology, National Institute of Genetic Engineering and Biotechnology, Iran; c Department of Clinical Biochemistry, Faculty of Medical Sciences, Tarbiat Modarres University, Iran; d Institute of Agricultural Biotechnology, National Institute of Genetic Engineering and Biotechnology, Iran

**Keywords:** Biotechnology, Molecular biology, Thermostable, *Cohnella* sp. A01, Cloning, Heterologous expression, Laccase, Bacteria, Bacterial genetics

## Abstract

Laccase (EC 1.10.3.2; benzenediol; oxygen oxidoreductases) is a multi-copper oxidase that catalyzes the oxidation of phenols, polyphenols, aromatic amines, and different non-phenolic substrates with concomitant reduction of O_2_ to H_2_O. Enzymatic oxidation techniques have the potential of implementation in different areas of industrial fields. In this study, the *Cohnella* sp. A01 laccase gene was cloned into pET-26 (b+) vector and was transformed to *E. coli* BL21. Then it was purified using His tag affinity (Ni sepharose resin) chromatography. The estimated molecular weight was approximately 60 kDa using SDS-PAGE. The highest enzyme activity and best pH for 2,6-dimethoxyphenol (DMP) oxidation were recorded as 8 at 90 °C respectively. The calculated half-life and kinetic values including K_m_, V_max_, turn over number (k_cat_), and catalytic efficiency (k_cat_/K_m_) of the enzyme were 106 min at 90 °C and 686 μM, 10.69 U/ml, 20.3 S^−^, and 0.029 s^−1^ μM^−1^, respectively. The DMP was available as the substrate in all the calculations. Enzyme activity enhanced in the presence of Cu^2+^, NaCl, SDS, n-hexane, Triton X-100, tween 20, and tween 80, significantly. The binding residues were predicted and mapped upon the modeled tertiary structure of identified laccase. The remaining activity and structural properties of *Cohnella* sp. A01 laccase in extreme conditions such as high temperatures and presence of metals, detergents, and organic solvents suggest the potential of this enzyme in biotechnological and industrial applications. This process has been patented in Iranian Intellectual Property Centre under License No: 91325.

## 1 Introduction

Laccase (benzodiol; oxygen oxidoreductase (EC.1.10.3.2)) is a multi-copper oxidase catalyzing the oxidation of phenols, poly-phenols, aromatic amines, and a variety of non-phenolic substrates [1]. The oxidation reaction happens through the Ping-Pong mechanism in which electron transference occurs in each step. The oxygen molecule (O_2_) first oxidizes the enzyme to produce the reactive intermediate (laccase-oxygen) along water; then laccase-oxygen removes one electron from the substrate [2]. This highly reactive substrate-driven radical is able to participate in a variety of reactions such as rearrangements and polymerizations. The initial laccase will be reformed through obtaining four dismissed electrons during oxygen reduction [3]. Laccase usually bears four copper atoms that are concentrated in three centers [4]. The type I copper has been suggested to have initial substrate oxidation role and its absence leads to enzyme inactivation. The copper type II is a single atom which binds to the nitrogen and oxygen donors to form a flat square. The type III contains two copper atoms that are responsible for oxygen reduction. The reduction of type I copper by an organic compound is the prerequisite of the oxygen attachment to type II/III coppers [3].

Laccase is naturally produced by numerous organisms such as plants, fungi, some insects, and bacteria. Its roles in a variety of cellular processes such as biosynthesis of pigments, spore production, pathogenicity, repairing the tissue damages, and lignin production and degradation were mainly investigated in fungal and plants [5], while there is a limited information of them in bacteria. The first bacterial laccase was identified in *Azospirillum lipoferum* with its important functions in production of cellular pigments, consumption of the plant phenolic compounds, and electron transfer [6]. The environment-friendly oxidation technologies has been directed to oxidizing enzymes [7] and manifold studies were dedicated to discover applicable kinds of laccase for various industrial purposes. This new area of interest was expanded so much so that in September 2014, laccase was called "the green alternative for chemical oxidation" [8] and promised the likeliness of finding other novel biocatalysts for answering the green chemistry needs and rules. Some of the industrial applications of laccase are delignification of pulp, textile bleaching, waste water treatment, production of detergents and bleaches, modification of biopolymers, development of biosensors (detection of various phenolic compounds and azides), and oxidizing agent in hair dyes [5].

The usual chemical oxidation techniques require toxic materials and produce non-specific reactions thus the biological oxidation techniques have attracted plenty of attention lately [9]. Due to the importance of thermostable laccase in biological enzymatic oxidation [10], the thermophilic bacterium *Cohnella* sp. A01 (PTCC No: 1921) laccase was investigated in the current study. The recombinant enzyme was exogenously expressed in *E. coli* BL21, purified and characterized biochemically and structurally to evaluate its capabilities in industrial processes, especially the textile industry.

## 2 Materials and methods

### 2.1 Materials

DNA purification kit was obtained from PEQLAB. PCR Purification Kit was purchased from Bioneer (Seoul, Korea). Plasmid purification kit was purchased from Roche (Basel, Switzerland). TAclone PCR Cloning Kit was provided by insta-clone kit. DNA ladder, Protein molecular weight markers, Taq polymerase, Pfu DNA Polymerase, T4 DNA ligase, *XhoI* and *NcoI* enzyme, IPTG, and X-GAL were purchased from Fermentas (Glen Burnie, MD, USA). Ampicillin, kanamycin, and 2,6-dimethylphenol (DMP) were obtained from Sigma (St. Louis, USA). *E. coli* DH5α and *E. coli* BL21 (DE3) strains, and vector pET-26b (+) were obtained from Invitrogen (Carlsbad, CA, USA). Ni Sepharose resin was purchased from Qiagen. All other chemicals were provided from Merck (Darmstadt, Germany).

### 2.2 Tertiary structure prediction, refinement, and validation

The Translate (http://web.expasy.org/translate) and ProtParam (https://web.expasy.org/protparam/) tools of Expasy server were used for translating the nucleotide sequence of the enzyme to amino acid sequence and physiochemical characterization, respectively. The three dimensional structure of the enzyme was modelled through I-TASSER server (https://zhanglab.ccmb.med.umich.edu/I-TASSER/). The energy minimization of the tertiary structure was performed by SPDBV v4.10 (GROMOS 96 as the applied algorithm) and other structural refinements were done by GalaxyRefine server (http://galaxy.seoklab.org/). To estimate the accuracy of predicted model before and after refinements, Ramachandran plot was analyzed by Rampage server (http://mordred.bioc.cam.ac.uk/∼rapper/rampage.php) and then its z-score was calculated by ProSA-Web server (https://prosa.services.came.sbg.ac.at/prosa.php).

### 2.3 Active site and Cu^2+^ binding sites prediction

The active site was predicted by RaptorX Binding server (http://raptorx.uchicago.edu/BindingSite/) according to the protein sequence. Then Cu^2+^ probable binding sites were predicted by IonCom server (https://zhanglab.ccmb.med.umich.edu/IonCom/). The provided data compared together to be validated and confirmed.

### 2.4 Cloning, expression and purification of *Cohnella* Sp. A01 Laccase

*Cohnella* sp. A01 was grown in nutrient broth medium at 60 °C and 180 rpm orbital shaking for 72 hours. The grown bacteria were used to extract genomic DNA. The forward (5′CTTCCATGGCGACGGACAATACGGACAACATGGAAG3′) and reverse (5′CTTCTCGAGCTCTGGCTTATTTCCCGCGTTC3′) primer pairs with *Nco*I and *Xho*I restriction sites (the underlined residues) were implemented to amplify the laccase gene (1600 bp) in pTZ57R/T cloning vector. Then it was sent to Genfanavaran Company for sequencing regarding M13 primer pairs. After confirming the validity of laccase sequence, the amplified gene was inserted between *Nco*I and *Xho*I restriction sites on pET-26b (+) as the expression vector. Eventually, the recombinant expression vector transferred into *E. coli* BL21. A single colony of the transformed bacteria was grown overnight in LB medium at 37 °C with 30 μg/mL of kanamycin then was transferred into a fresh LB medium. In order to induce the protein expression and appropriate folding, 1 mM IPTG and 0.5 mM CuSO_4_ were added to the inoculated media, after reaching 0.6 as the desired OD_600_. The bacterial cells were pelleted (4 °C, 20 minutes, 4000 ×g) after 16 hours incubation at 37 °C. The precipitate of bacteria was resuspended in lysis buffer (50 mM NaH_2_PO_4_, 300 mM NaCl, 10 mM Imidazole, pH = 8). The resulted suspension was subjected to ultrasonic waves (UP400S - Hielscher Ultrasonics GmbH) incubating on ice for four sets of one minute pulses (70 mHz) with 20s intervals in order to break the cells' walls. The suspension was subsequently centrifuged (4 °C, 30 minutes, 9000 ×g). The supernatant was collected to be used for protein expression analysis and purification. The negative control was designed as same as previous sections except having the empty pET-26b (+) vector in *E. coli* BL21 instead of recombinant one. Laccase was designed to have His-tagged to allow its better purification through affinity chromatography using Nickel-sepharose column; the resulted fractions were visualized by SDS-PAGE. The purified enzyme was dialyzed against phosphate buffer (10 mM K_2_HPO_4_, pH = 8) and its concentration was estimated through Bradford's assay.

### 2.5 Enzyme activity assay

The dialyzed laccase activity was measured based on its ability to oxidase DMP to 3,3′,5,5' –tetramethoxy-biphenyl-4,4' –diol [11]. The laccase activity was monitored in the presence of 4 mM DMP at 470 nm (Beckman DU 530 from US). The calculations were performed according to A = ɛcl (ɛ = 14,800 M^−1^cm^−1^) formula [12]. The phosphate buffer (10 mM K_2_HPO_4_) in the presence of DMP was used as the control reaction.

### 2.6 Kinetic study

DMP concentrations of 0.3, 0.6, 1.25, and 2.5 mM were used to examine the kinetic properties of the laccase. Michaelis-Menton plot was used to calculate the values of V_m_ and K_m_ in GraphPad Prism 6.07. The K_m_ value represents the enzyme affinity towards the substrate; the lower K_m_ corresponds to higher affinity. The catalytic functionality can be considered as a criterion for determining the enzyme's industrial applicability.

### 2.7 The effect of temperature and pH on the enzyme's activity and stability

For determining the optimum temperature, the laccase activity was measured after incubation for 30 minutes at 20–100 °C. The optimum pH then was found by measuring the enzyme activity at 90 °C incubating with 50 mM substrate solutions in pH 3–10 (citrate phosphate buffer for pH 3–7, phosphate buffer for pH 6–8, glycine-sodium hydroxide for pH 9–10) [13].

The enzyme thermostability was determined by measuring the enzyme activity after 10, 20, 30, 40, 50, 60, 90, and 120 minutes incubation in 60, 90, and 100 °C; and then incubation at optimal condition (pH = 8 and 90 °C) for 30 minutes. It was then confirmed by measuring the enzyme stability in the temperature range of 10–100 °C for 90 minutes and incubation at optimal condition (pH = 8 and 90 °C) for 30 minutes.

The pH stability of the enzyme was first measured by measuring the enzyme activity in different pH ranges [5, 6, 9, 10] in four subsequent time sections with one-hour intervals and incubation at optimal condition (pH = 8 and 90 °C) for 30 minutes; then as a confirming test, the enzyme was incubated at pH 3 to 10 for 90 minutes and then incubation at optimal condition (pH = 8 and 90 °C) for 30 minutes.

### 2.8 The effect of metal ions, organic solvents, inhibitors, surfactants, and SDS on laccase activity

The enzyme activity was screened under the effect of 1 and 5 mM metal ions, including Cu^2+^, Na^+^, Ni^2+^, Zn^2+^, Fe^2+^, and Mn^2+^. The effect of organic solvents such as acetone, glycerol, methanol, ethanol, isopropanol, isobutanol, N-hexane, and acetic acid with concentrations of 25 and 50% (v/v) was also evaluated. The 1 and 5mM of β mercaptoethanol, PMSF, cysteine, EDTA, and NaN_3_ as the inhibitors were also used for the same purpose. Tween 20, tween 80, and triton X100 with the concentrations of 0.1, 0.5, and 5 mM and SDS with the concentrations of 1.56, 3.12, 6.25, 12.5, 25, and 50 mM (as surfactants) were added to the enzyme's activity reactions to analyze their effects. All reactions were treated for 1 hour and the activity was determined at 90 °C and pH 8. The activity of the untreated enzyme was considered as the control.

### 2.9 Extrinsic and Intrinsic Fluorescence

Laccase (100 μgr/ml) was incubated with SDS (25 and 50 mM) at 90 °C within the pH range of 3–10 (citrate phosphate buffer for pH 3–7, phosphate buffer for pH 6–8, glycine-sodium hydroxide for pH 9–10, 50mM) for 60 minutes to perform the fluorescence experiments using spectrophotometer (Varian Cary Eclipse Fluorescence Spectrophotometer, Santa Clara, USA).

The 8-Anilinonaphthalene-1-sulfonic acid (ANS) with the final concentration of 40 μM was added to the enzyme and then incubated at 25 °C for 10 minutes. The samples were excited at 360 nm and emission spectra were recorded from 400-600 nm. The spectral bandwidth was set at 10 nm. To perform the intrinsic fluorescence experiment, the samples were excited at 280 nm and emission spectra were recorded from 300-400 nm. The spectral bandwidth was set at 5 nm. The fluorescence graphs were plotted using SigmaPlot v12.5. Stored laccase in phosphate buffer (10 mM K_2_HPO_4_, pH = 8) at 4 °C was used as control.

### 2.10 Circular dichroism (CD)

Laccase (100 μgr/ml) was incubated in two separate vials at 90 °C and with 25 mM SDS for 60 minutes. Then its secondary structure composition was recorded in the 180–240 nm wavelength range. The far UV circular dichroism (CD) spectroscopy was analyzed with spectropolarimeter 158 J-715 (Jasco, Tokyo, Japan). Stored laccase in phosphate buffer (10 mM K_2_HPO_4_, pH = 8) at 4 °C was used as control.

## 3 Result

### 3.1 Cloning, expression, purification, and kinetic properties calculation of *Cohnella* sp. A01 laccase

**Fig. 1:**
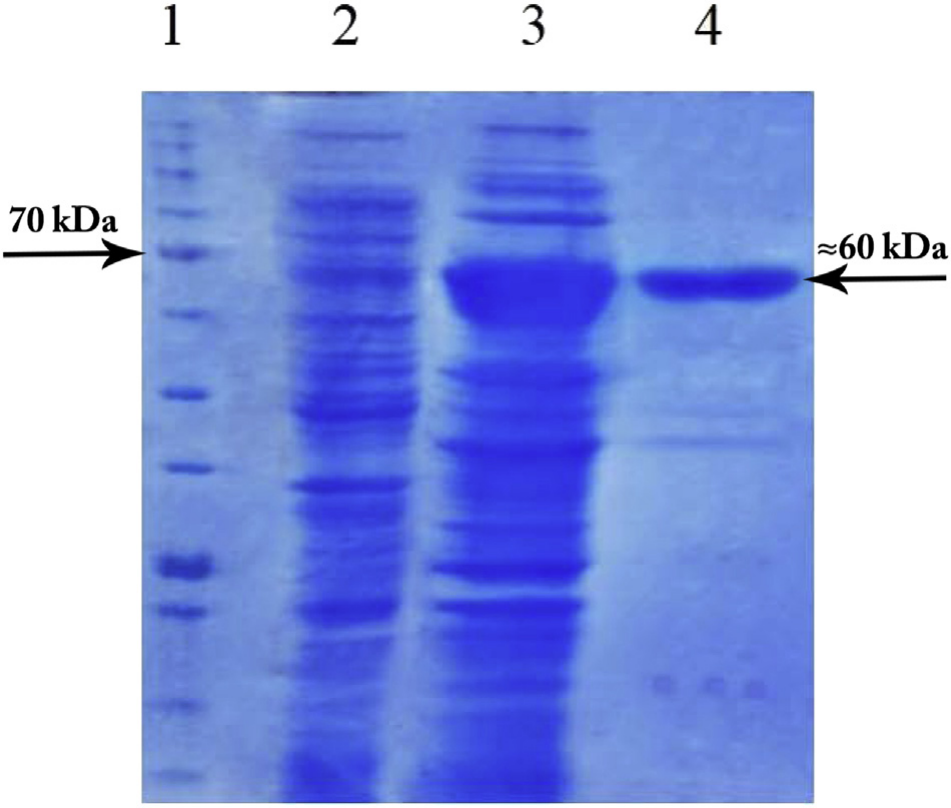
Heterologous expression and purification of *Cohnella* Sp. A01 laccase. SDS–PAGE of the expressed and purified *Cohnella* Sp. A01 laccase [1]. Protein marker [2], The total protein before induction with IPTG as the negative control [3], The total protein after induction with IPTG [4], The purified laccase (Compelete file: Supplementary Fig. S1).

The alignment of amino acid sequence of *Cohnella* sp. A01 laccase with the protein sequences of Genbank revealed similarity to other related proteins; the most similar protein was *Paenibacillus darwinianus* laccase (Accession: EXX87833.1) with 68% identity. The laccase gene was amplified by PCR and then cloned in pTZ57R/T vector. The cloning was confirmed by sequencing. After confirming the gene sequencing, *Cohnella* sp. A01 laccase with the accession number of AKL79441.1 was recorded in GenBank database. Laccase gene was subcloned into pET-26b (+) as an expression vector and transferred into *E. coli* BL21 strains in order to be over expressed. The heterologous expression of the *Cohnella* sp. A01 laccase in *E. coli* BL21 was confirmed through SDS-PAGE analysis. The purity of the enzyme was examined and shown as a single band on acrylamide gel with an approximate size of 60 kDa by SDS PAGE after Ni Sepharose resin purification. The results are shown in Fig. 1. Yield of purification, purification fold, and specific activity of the purified enzyme were found to be 33.3, 4.7, and 20 (U.mg^−1^), respectively. The kinetic properties of the purified enzyme were assessed through calculating the values of K_m_, V_max_, turn over number (k_cat_), and catalytic efficiency (k_cat_/K_m_) in the presence of DMP. The values were respectively 686 μM, 10.69 U/ml, 20.3 S^−^, and 0.029 s^−1^ μM^−1^. The results of *Cohnella* sp. A01 laccase purification profile and kinetic parameters were summarized in Table 1.

**Table 1:** Purification profile and kinetic parameters of the *Cohnella* sp. A01 laccase.

Steps	Volume (ml)	Protein (mg/ml)	Total protein (mg)	Total activity (units)	Specific activity (units/mg)	Purification fold	Yield (%)	K_m__(_μM)	V_max_ (U/ml)	k_cat_ (S^−^)	catalytic efficiency (S^−1^ μM^1^)
Crude	4	1.4	5.6	24	4.2	1	100	-	-	-	-
1	4	0.1	0.4	8	20	4.7	33.3	686	10.69	20.3	0.029

### 3.2 Evaluation of temperature and pH on the laccase activity and stability

The optimum temperature of this enzyme was estimated to be 90 °C with a half-life of 1 hour and 46 minutes. The enzyme conserved more than 60% of its activity at 100 °C. The thermostability of the enzyme was approved since it maintained more than 60% of its activity after 90 minutes incubation at 60 to 90 °C. The enzyme still conserved 48% of its activity after the same duration of incubation at 100 °C.

**Fig. 2:**
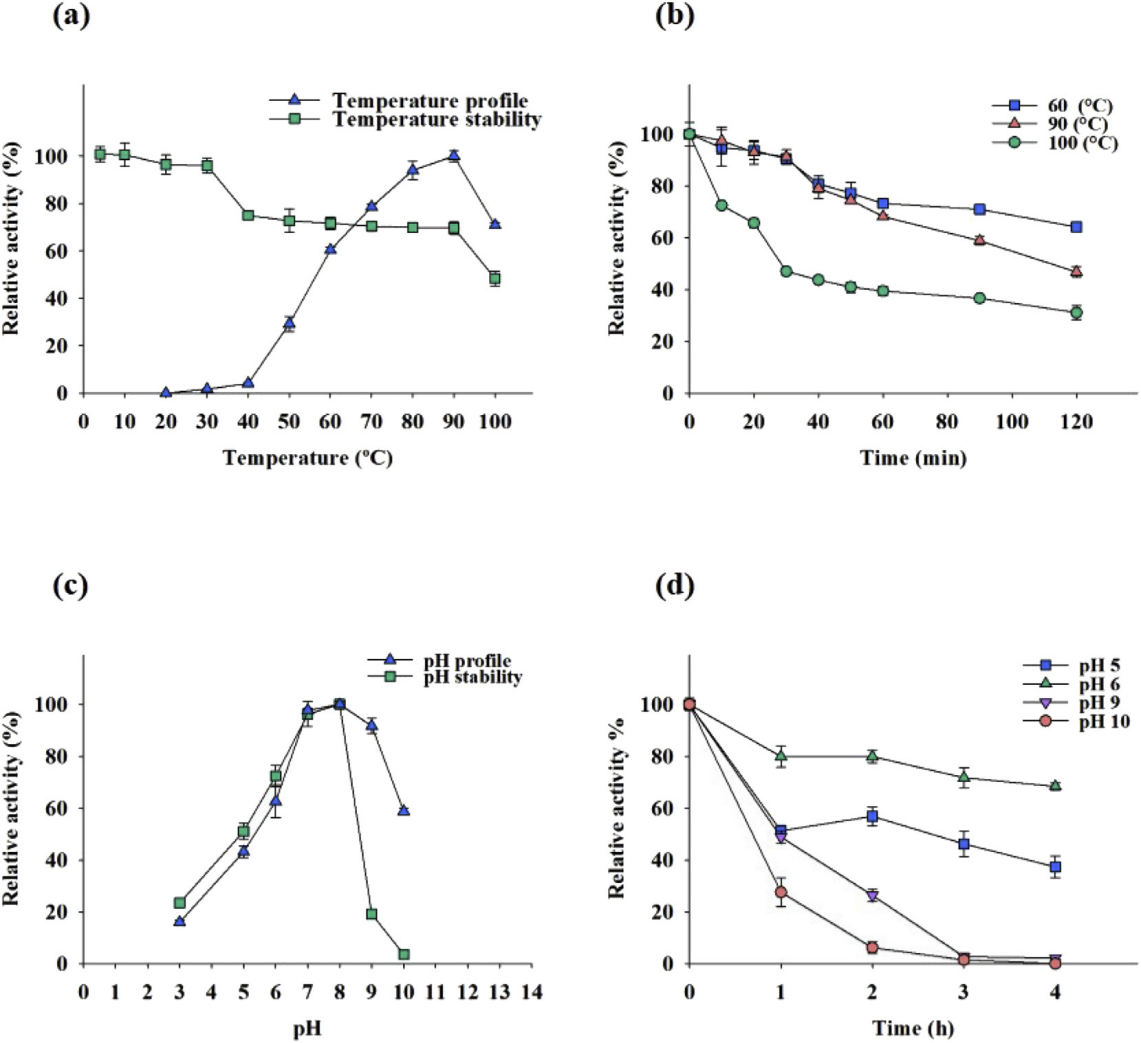
The effect of pH and temperature on the *Cohnella* sp. A01 laccase. (a) Temperature activity and stability. (b) Temperature stability. (c) pH activity and stability. (d) pH stability.

The optimum pH of the *Cohnella* sp. A01 laccase was found to be 8. The relative activities of the enzyme were 62%, 97%, 91%, and 58% in the pH range of 6, 7, 9, and 10. The pH stability of the enzyme was analyzed and approved in the pH range of 5, 6, 7, and 8 regarding maintaining more than 50% of its activity after 90 minutes incubation. The results are shown in Fig. 2.

### 3.3 Evaluation of metal ions, inhibitors, organic solvents, and surfactants effects on the laccase activity

**Fig. 3:**
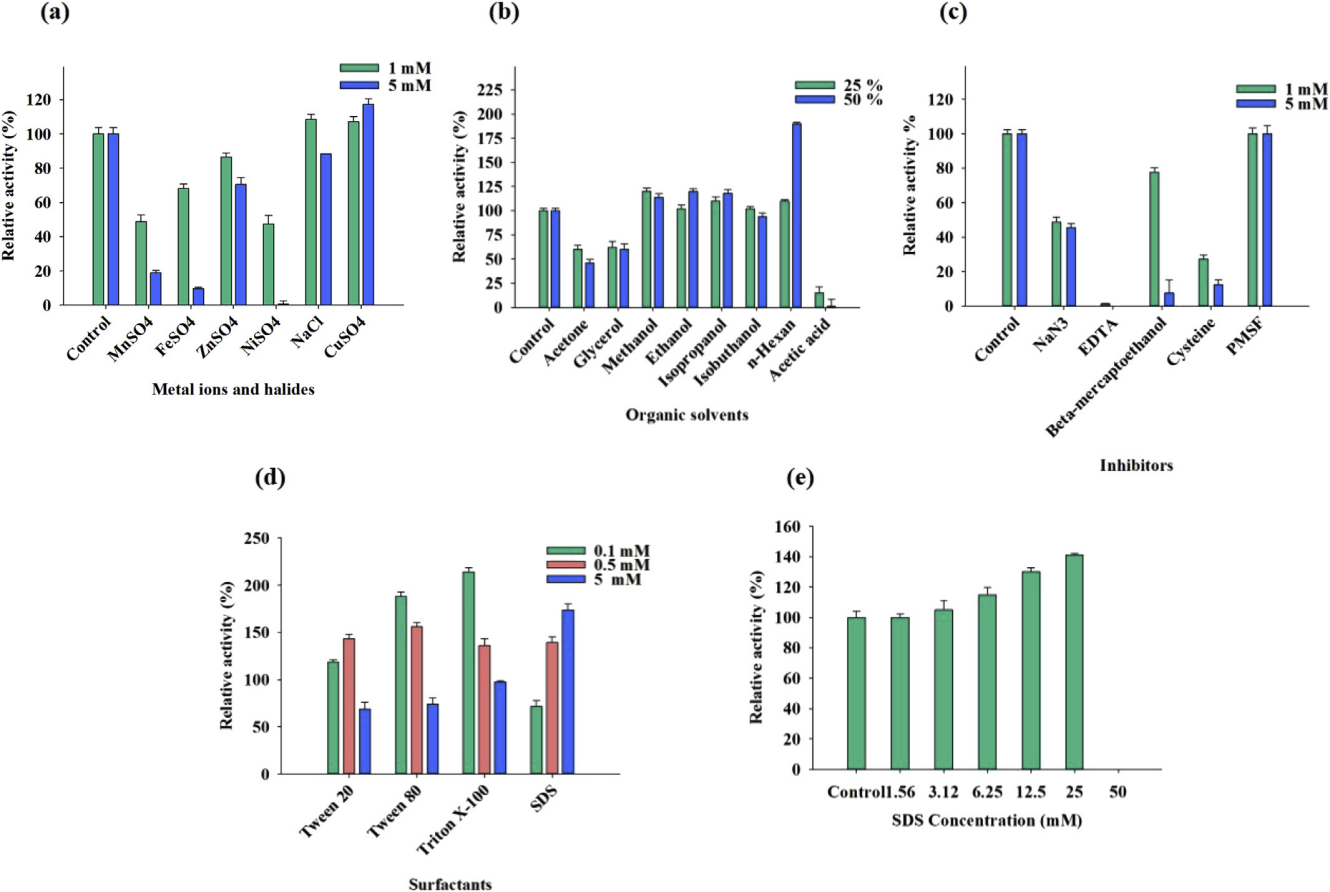
The effect of some organic solvents, metal ions/halides, inhibitors, and surfactants on the *Cohnella* sp. A01 laccase. (a) Metal ions/halides (b) organic solvents (c) inhibitors (d) surfactants (e) SDS. The laccase activity was analyzed at 90 °C and pH 8 by triple repeats.

The examined effects of metal ions including Cu^2+^, Na^+^, Ni^2+^, Zn^2+^, Fe^2+^, and Mn^2+^ on the enzyme activity are presented in Fig. 3a. According to the results, 1 and 5 mM of Cu^2+^ and 1 mM of Na^+^ increased the activity and 1 mM of Zn^2+^, Fe^2+^, Mn^2+^ and Ni^2+^ reduced this value to 86, 68%, 48%, and 47%, respectively, considering the untreated enzyme as control (Fig. 3a).

The evaluated effects of organic solvents at concentrations of 25 and 50% (v/v) on *Cohnella* sp. A01 laccase activity are represented in Fig. 3b. The enzyme activity was increased 100% following 50% N-hexane treatment. The approximate lowered activity to 50% was observed following 25% and 50% acetone and glycerol treatments.

The inhibitors effects (1 and 5 mM of each inhibitor) in comparison with control reaction are provided in Fig. 3c. The EDTA inhibited most of enzyme activity and NaN_3_ reduced the activity to 48% and 45%, respectively. The Cysteine dampened the enzyme activity to 27% and 12%, respectively. The activity of this enzyme was lowered to 77 and 7% when 1 and 5 mM β mercaptoethanol was added).

The Fig. 3d represents the surfactants' effects on enzyme activity. The concentrations of 0.5 mM Tween 20, 0.1 mM Tween 80, and 0.1 mM Triton X100 increased the enzyme activity approximately 50%, 100%, and 100%, respectively. Nevertheless, the 5 mM concentration of the mentioned compounds decreased the activity to less than 50%. There was a surprising 41% increase in the enzyme activity under treatment of up to 25 mM SDS (Fig. 3e).

### 3.4 Fluorescence experiments

The laccase conformational changes were investigated under the influence of temperature and pH through intrinsic fluorescence. The fluorescence intensity was decreased in acidic condition (pH: 3, 5, 6), while similar fluorescence intensities were observed in the pH range of 7–10. The data at pH of 4 (assumed to be the enzyme's pI) is not reliable due to the protein precipitation.

**Fig. 4:**
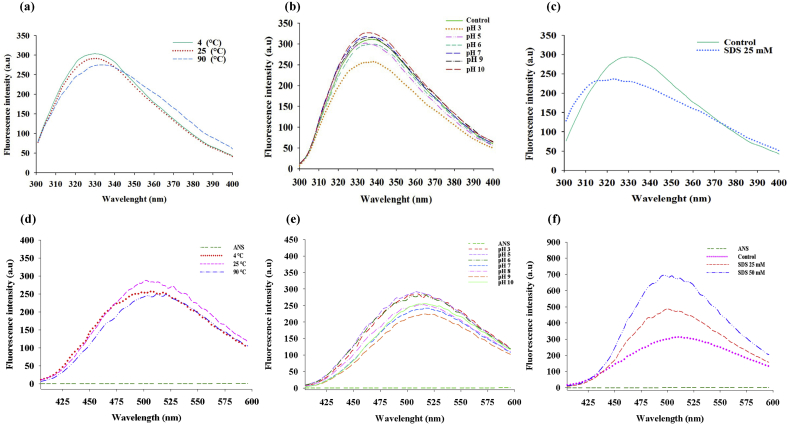
Extrinsic and Intrinsic Fluorescence. (a) The analyzed intrinsic fluorescence at the temperature of 4, 25, and 90 °C. (b) The analyzed intrinsic fluorescence at the pH range of 3–10. (c) The analyzed intrinsic fluorescence in the presence of SDS. (d) The analyzed extrinsic fluorescence at the temperature of 4, 25, and 90 °C. (e) The analyzed extrinsic fluorescence at the pH range of 3–10. (f) The analyzed extrinsic fluorescence in the presence of SDS.

The laccase structural changes following exposure to different pH ranges in compare to untreated enzyme were evaluated through extrinsic fluorescence technique utilizing ANS. The fluorescence intensity was increased within the pH range of 3–6 and maintained constant within the pH range of 7–10. The fluorescence intensity was increased in the presence of 25 mM SDS (Fig. 4).

### 3.5 Circular dichroism (CD)

**Fig. 5:**
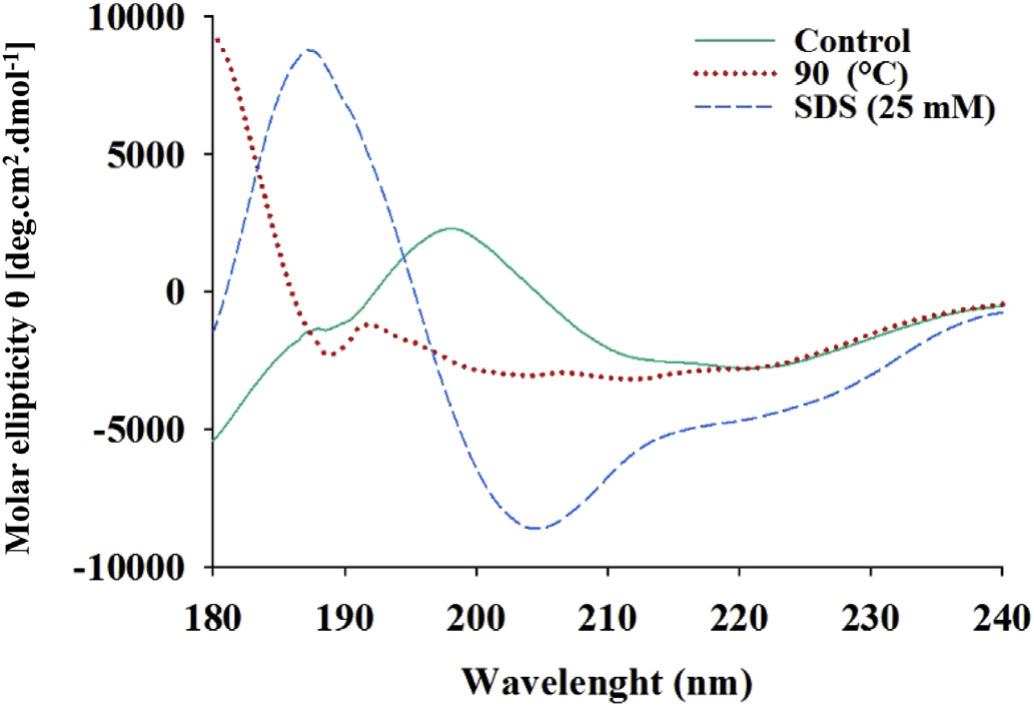
The secondary structure evaluation by Circular Dichroism (CD) spectroscopy. The *Cohnella* sp. A01 laccase secondary structure was evaluated at 90 °C and 25mM SDS. The control sample is the stored laccase in 4 °C in phosphate buffer (10mM K_2_HPO_4_, pH = 8).

Analysis of the laccase secondary structure through Circular Dichroism spectra showed that it contains 7.4 % α-helix, 66% β-sheet, 7.5 % β-turn, and 18.8 % loop (random coil). The β-sheets were remained almost unchanged (67.2 %) at 90 °C, while the α-helix and β-turn were reduced to 2.2 % and 3.8 %, respectively. The amount of loops were increased to 26.8 %. In the presence of 25 mM SDS, the amount of α-helix reached zero and decreasing and increasing in the number of β-sheets and β-turns were observed, respectively (Fig. 5 and Table 2).

**Table 2:** The secondary structure compartments of *Cohnella* sp. A01 laccase through CD spectroscopy.

	Helix (%)	Beta sheet (%)	Turn (%)	Loop (%)	RMS
CNT	7.4%	66.3%	7.5%	18.8%	122.74
TEMP 90	2.2%	67.2%	3.8%	26.8%	67.485
SDS 25	6.0%	57.4%	0.0%	36.7%	27.980

### 3.6 Tertiary structure prediction, refinement, and validation

**Fig. 6:**
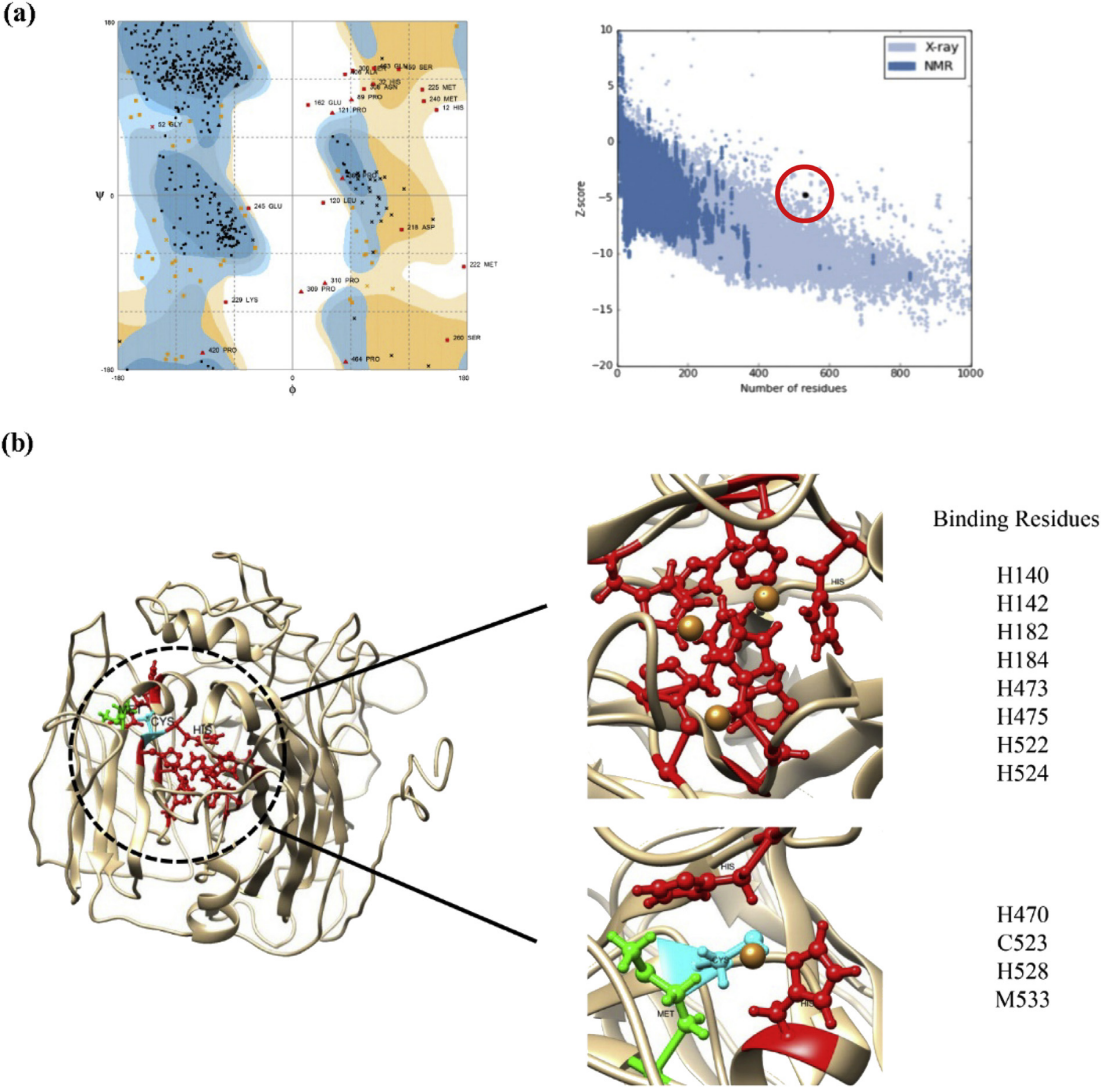
Tertiary Structure Validation and Binding Pockets Mapping. (a) The Ramachandran and z-score plots of *Cohnella* sp. A01 laccase tertiary structure after refinements, resulted from RAMPAGE and ProSA-Web servers, respectively. The Ramachandran plot shows the residues in most favored (86.6%), allowed (8.9%) and outlier regions (4.5%). The z-score (-4.75) represents the resemblance between predicted tertiary structure and native proteins. The black dot is the predicted model. The scores approves the authenticity of the predicted model. (b) The tertiary structure of *Cohnella* sp. A01 laccase that was predicted by I_TASSER and visualized by Chimera 1.11.2. The active sites were predicted by RaptorX Binding server with 6.41e-17 as p-value and 290.9(60.6) as uGDT (GDT) score that both indicate high quality model. The binding residues, according to the predicted model, is provided by the first letter of them. The first pocket contains eight Histidines that can bear three Cu^2+^ ions according to homologous laccases in previous studies. The, second pocket contains two Histidines, one Methionine and one Cysteine that can bear one Cu^2+^ ion. The Histidine, Cysteine and Methionine are shown in red, cyan and green, respectively. The Cu^2+^ ions are shown in golden spheres. The pose of interactions between Cu^2+^ and surrounded residues is hypothetical.

According to ProtParam tool of Expasy, the laccase contains 557 amino acids and approximate molecular weight of 61.59 kDa. Among five predicted models resulted from I-TASSER, the first model by C-score = -0.83, TM-score = 0.61 ± 0.14, and RMSD = 9.4 ± 4.6Å was counted as the reliable one. The model was given to GalaxyRefine server for structural refinements and then experienced energy minimizations by SPDBV v4.10 software. The z-scores by ProSA-Web server were -2.97 and -4.75 before and after improvements, respectively. Although both values indicated the resemblance of the predicted model to native proteins but the improved value of -4.75 after refinements indicated the necessity of this step. The Ramachandran Plot which indicates the percentage of residues in favored, allowed, and disallowed regions resulted in respective values of 66.7%, 20.3%, and 13.0% before and 86.6% 8.9%, and 4.5% after structural improvements. The percentage of amino acids exist in the favored and allowed region were improved and meet 95.5% and is confident (Fig. 6a).

### 3.7 Active site and Cu^2+^ binding sites prediction

The predicted Cu^2+^ binding sites by IonCom server were Histidine (H) 140, H142, Tryptophan (W) 180, H182, H184, Asparagine (N) 426, H470, H473, H475, H477, Glutamate (E) 506, D514, H522, Cysteine (C) 523, H524, D525, H528, and Methionine (M) 533. There were three predicted binding regions from RaptorX Binding server that two of them specifically comprise Cu^2+^ ions. The first region includes Histidine (in 140, 142, 182, 184, 473, 475, 522, 524 residues) and the second one includes Histidine (in 470 and 528 residues), Cysteine 523, and Methionine 533. The resulted data were compared and confirmed each other (Fig. 6b).

## 4 Discussion

The *Cohnella* sp. A01 laccase was found to be thermophilic and thermostable, the main prerequisites for an industrial enzyme [14]. The high temperature increases the reaction rate through cutting down the reaction time and preventing the reactions contamination [15]. The comparison of optimum temperature and stability of *Cohnella* sp. A01 laccase and other kind of heterologously expressed bacterial laccase are shown in Table 3 [16, 17, 18, 19, 20, 21, 22, 23, 24, 25, 26, 27, 28, 29, 30, 31].

**Table 3:** The comparison of optimum temperature and stability of *Cohnella* sp. A01 laccase and other kind of heterologously expressed bacterial laccase.

Origin	Host	Optimum temperature (°C)	Temperature stability	Reference
*Cohnella* sp. A01	*E. coli* BL21	90	60%/100 °C/pH 8/90 min	This study
*Bacillus vallismortis* fmb-103	*E. coli* BL21	84	50%/70 °C/pH 4.8/10 h	[16]
*Bacillus subtilis*	*E. coli* DH5a	30	-	[17]
*Meiothermus ruber* DSM 1279	*E. coli* DH5a	70	50%/60 °C/120 min	[18]
*Paenibacillus glucanolyticus*SLM1	*E. coli* BL21 (kDE3)	40	76%/60 °C/pH 7.0/4 h	[19]
*Bacillus coagulans* LMCO	*E. coli*	30	>80%/70 °C/30 min	[20]
*Thermus thermophilus* SG0.5JP17-16	*Pichia pastoris*	90	>75%/80 °C/4 h	[21]
*P. ananatis*	*E. coli*	30–50	>40%/60 °C/10 min/pH 2.5	[22]
*B. pumilus* strain W3	*E. coli*	50	NR	[23]
*B. licheniformis*	*Pichia pastoris*	70	50%/70 °C/6.9 h	[24]
Uncultured Bacterium lac21	*E. coli*	45	>60%/35 °C–50 °C	[25]
*Ochrobactrum.*sp.531	*E. coli*	37	-	[26]
*B. pumilus* DSM 27	*E. coli*	37	-	[27]
*B. licheniformis*	*E. coli*	37	-	[28]
*Aeromonas hydrophila* WL-11	*E. coli*	37	40%/70 °C/10 min	[29]
*Streptomyces ipomoea* CECT 3341	*E. coli*	37	-	[30]
*B. subtilis*	*E. coli*	75	50%/80 °C/112 min	[31]

Identification of the optimum pH is needed to manage the enzyme's purification buffers, storage, and industrial applications [32]. The *Cohnella* sp. A01 laccase had the optimum pH of 8 and it was appropriately stable in the pH range of 5–8. The optimum pH of laccase depends strictly on the implemented substrate, oxygen molecules, or its origin. The reductive potential between the substrate and the type I copper and then hydroxide anion attachment to the copper types II and III is an important factor in determining the optimum pH [33]. Table 4 shows the comparison of optimum pH of *Cohnella* sp. A01 laccase and other reported laccases in the presence of DMP substrate [34, 35, 36, 37, 38, 39, 40].

**Table 4:** The comparison of optimum pH of *Cohnella* sp. A01 laccase and other reported laccases in the presence of DMP substrate.

Optimum pH	Microorganism	Reference
8	*Cohenlla* sp. A01	This study
3	*Trametes trogii*	[34]
3.5	*Pycnoporus* sp. SYBC-L1	[35]
3.5	*Pycnoporus* sp. Sybc-l1 Lac1	[36]
7	*Bacillus licheniformis*-CotA	[37]
3	*Physisporinus rivulosus Lac-3.5*	[38]
5.5	*Agaricus blazei*	[39]
6	*Pleurotus Sajor-caju*	[40]

Metal ions are crucial factors for enzymes' structure and function. The stability of the enzyme in the presence of metal ions could be an asset considering its use in industry such as pulp and paper and bioremediation [41]. The reduction of laccase activity in the presence of metal ions could be explained by the possible attachments of these ions to the copper types II and III that interfere with the internal electron transferring process affecting its activity [42]. For example, the activity reduction in the presence of Fe^2+^ may be due to its reactions with the laccase electron transferring system [43]. The enhanced enzyme activity in the presence of some ions, especially copper, can be observed in the majority of laccases. During the expression of laccase, the unsaturated Cu^2+^ residues lead to partial activation of the enzyme, hence reactivating process can be reached by Cu^2+^ ions treatments. This procedure has been successful for improving the activity of *Cohnella* sp. A01 laccase, in line with previous studies [44].

According to our results, EDTA approximately inhibited most of the enzyme activity since it binds to type II copper ions. Its attachment to unstable type II copper ions facilitates their collection and in following inhibits the enzyme activity more than before [34]. The decreased activity of laccase that was exposed to NaN_3_ may be due to electron transferring blockage in the types II and III copper binding sites [45]. Cysteine counts as a competitive inhibitor for polyphenol oxidases so the resulted decrease following Cysteine presence is due to this criterion. This amino acid binds to the enzyme's active site forming the enzyme-cysteine complex and reduces its activity [46]. The reduced enzyme's activity in the presence of β mercaptoethanol may be related to the reduction of the oxidized substrate by the sulfhydryl group [41].

Using the organic solvents instead of natural aqueous environment of the enzyme could increase its applicability in industries. In fact the organic solvents alter the activity of the enzymes through various physicochemical interactions. It has been demonstrated in some cases that an impossible enzymatic reaction in water could be performed in organic solvents [47]. In addition, the majority of laccase substrates are insoluble in water and increasing their solubility in the presence of organic solvents and their concentrations could enhance the enzymatic reaction rate [9]. On the other hand, the organic solvents are able to denature the proteins through removing the water molecules around them which are necessary for the functional groups [48]. Thus, the activity and stability of enzymes in such solvents could be of high importance for their applications in industry. According to the obtained results, enzyme activity was doubled in presence of n-hexane assuring the usage of laccase in textile waste water treatment where n-hexane is abundantly available. According to the previous studies, there is positive relation between the enzyme thermostability and stability in the presence of organic solvents. Furthermore, the halophilic enzymes count as potential biocatalysts in aqueous–organic media due to the similarity between organic solvents and salt (both reduce water activity) [49]. Regarding the stability of *Cohnella* sp. A01 laccase against temperature and salt, it is expected to be stable against organic solvents too. It has been reported that 10, 30 and 50 % aceton treatment increased the laccase activity 137, 89 and 35%, respectively [50].

Surfactants can facilitate the elimination of hydrophobic contaminants such as PAHs (Polycyclic aromatic hydrocarbons) through increasing the solubility, bioaccessibility, and emulsifyablity of hydrocarbons [51]. One application of laccase is the elimination of multi-cyclic aromatic hydrocarbons and xenobiotic compounds (the main contaminants of soil) [52]. SDS is an anionic detergent which disrupts almost all the noncovalent interactions in the proteins and increase the enzyme activity by its denaturing properties [53]. By exposing the *Cohnella* sp. A01 laccase to up to 25mM SDS, its activity enhanced by 41 percent which is impressive in comparison with other studies. For instance, in a study held by Badooei et al, the laccase activity was increased 4.3 fold when exposing to 0.05 mM SDS [53]. Increasing the enzyme activity was also observed in *Azospirillum lipoferum* laccase, while the inactivated laccase was reactivated by adding SDS and maximum activity was obtained up to 0.4 mM SDS [6]. Similarly, Ming-Qiang et al, showed that the activity of a fungus thermostable laccase was increased to 1.4% in 1mM SDS presence and decreased to approximately 50% in 5mM concentration of the compound [34]. The laccase from *Pycnoporus* sp. SYBC-L1 in 1 and 5 mM SDS had zero activity [36].

Decrease in the intrinsic fluorescence intensity in acidic conditions indicates the relative changes of enzymes' structures. The observed tiny redshifts in intrinsic fluorescence while studying the effect of temperature on laccase structure may relates to degradation of the enzyme's structure at 90 °C. SDS can decrease the enzyme's structural rigidity, improve its flexibility, and in following increase its activity so we observed the increased emission of extrinsic fluorescence in treated *Cohnella* sp. A01 laccase with 25 mM SDS.

The unique features of *Cohnella* sp. A01 laccase can be confirmed with screening the enzyme's secondary structure exposing to 25 mM SDS. As it was obtained through performing laccase Circular Dichroism, the amount of α-helix was reached to zero while decreasing and increasing in the amount of β-sheets and turns were observed, respectively. This can be due to conformational changes and flexibility enhancement of enzyme that lead to improvements in enzyme activity.

## 5 Conclusion

The laccase capabilities in oxidizing phenolic and nonphenolic compounds have made it a valuable enzyme for safe oxidizing technologies. The *Cohnella* sp. A01 laccase was exogenously expressed in bacterial host, purified, and characterized for its biochemical, thermodynamic, and structural features in the present study. This enzyme was naturally resistant to high temperatures, a wide pH range, and the presence of ions, surfactants, and organic solvents. The *Cohnella* sp. A01 laccase can be a candidate for industrial applications due to its high thermostability and maintained activity in harsh conditions. With reference to its distinctive features and the possibility of its production optimization during scale up studies, it can be a suitable alternative for the currently used laccases. This process has been patented in Iranian Intellectual Property Centre under Licence No: 91325.

## Declarations

### Author contribution statement

Masoomeh shafiee: performed the experiments; analyzed and interpreted the data; wrote the paper.

Farzaneh Afzali: analyzed and interpreted the data; wrote the paper.

Ali Ashgar Karkhane: analyzed and interpreted the data; wrote the paper.

Mehdi Ebrahimi: contributed reagents, materials, analysis tools or data.

Kamahldin Haghbeen: analyzed and interpreted the data.

Saeed Aminzadeh: conceived and designed the experiments; wrote the paper.

### Funding statement

This research did not receive any specific grant from funding agencies in the public, commercial, or not-for-profit sectors.

### Competing interest statement

The authors declare no conflict of interest.

### Additional information

No additional information is available for this paper.
