# Supplementary material for: Cohnella sp. A01 laccase: thermostable, detergent resistant, anti-environmental and industrial pollutants enzyme
Source: Heliyon. 2019 Sep 30;5(9):e02543. doi: 10.1016/j.heliyon.2019.e02543 (PMC6819783; doi:10.1016/j.heliyon.2019.e02543)

**Supplementary Figure Legends**

**Supplementary Figure S1:** **Heterologous expression and purification of *Cohnella* Sp. A01 laccase. SDS–PAGE of the expressed and purified *Cohnella* Sp. A01 laccase**. (1) Protein marker and the total protein before induction with IPTG as the negative control and the total protein after induction with IPTG, (2) The purified laccase, (3) cropped, sliced and combined image.

(1)


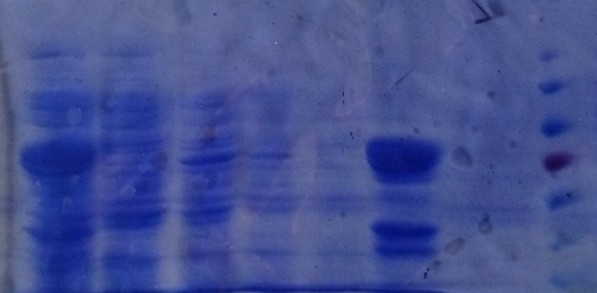


(2)


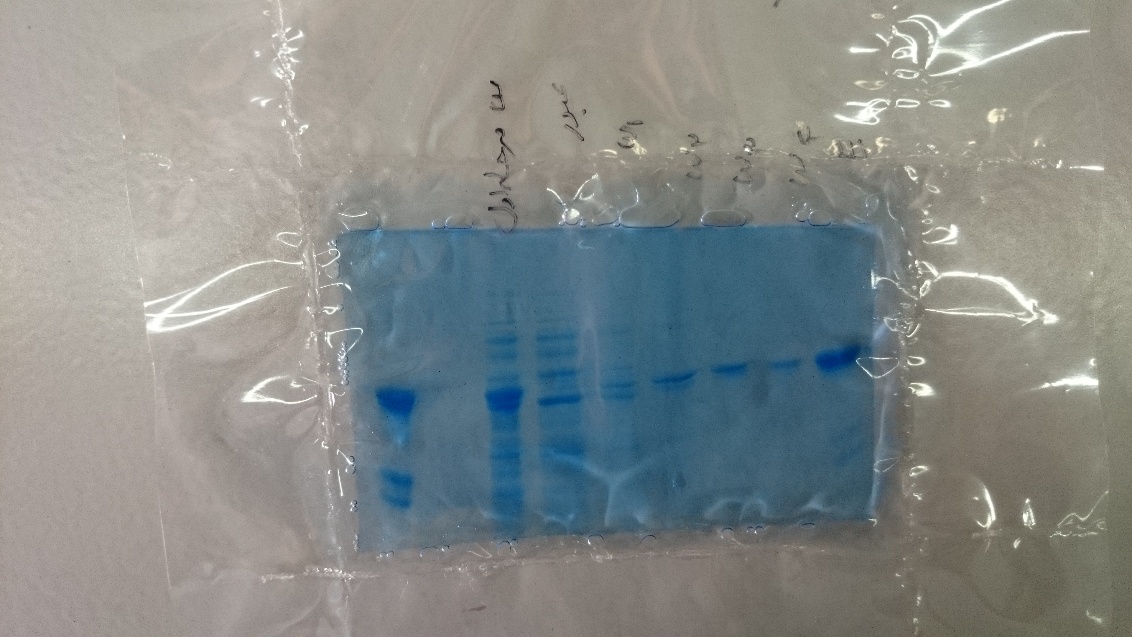


(3)


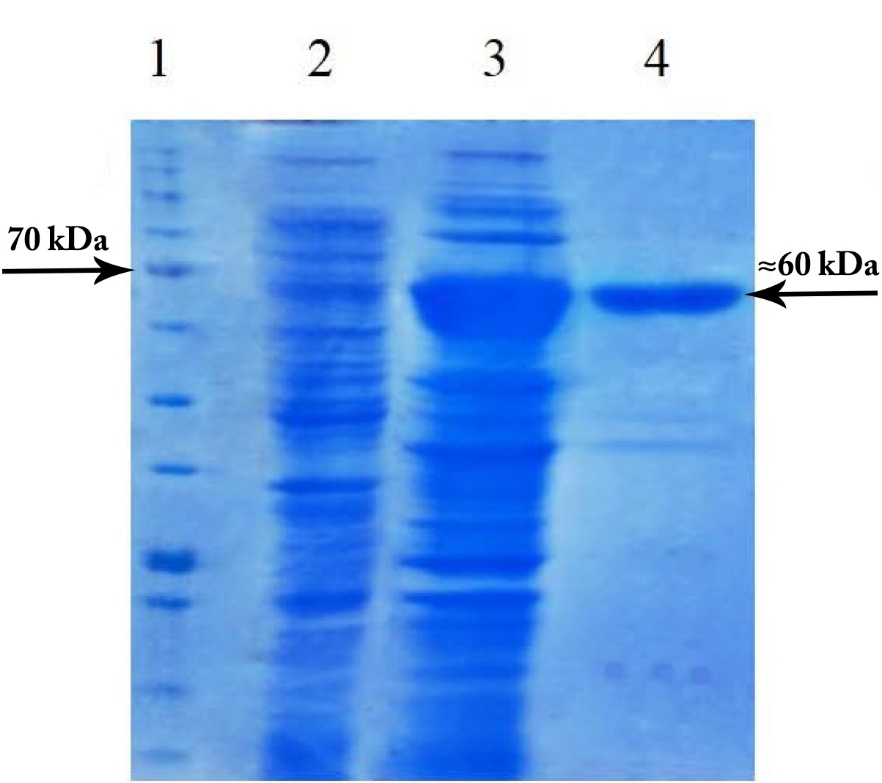

Supplement: supplementary 98 6 21 [file mmc1.docx]
